# Can Endothelin-1 Help Address the Diagnostic and Prognostic Challenges in Multimorbid Acute Heart Failure Patients?

**DOI:** 10.3390/life15040628

**Published:** 2025-04-09

**Authors:** Bianca-Ana Dmour, Minerva Codruta Badescu, Cristina Tuchiluș, Corina Maria Cianga, Daniela Constantinescu, Nicoleta Dima, Ștefania Teodora Duca, Awad Dmour, Alexandru Dan Costache, Maria-Ruxandra Cepoi, Adrian Crișan, Sabina Andreea Leancă, Cătălin Loghin, Ionela-Lăcrămioara Șerban, Irina Iuliana Costache-Enache

**Affiliations:** 1 Department of Internal Medicine, “Grigore T. Popa” University of Medicine and Pharmacy, 16 University Street, 700115 Iasi, Romania; bianca-ana.gherasim-dmour@d.umfiasi.ro (B.-A.D.); nicoleta.dima@umfiasi.ro (N.D.); stefania-teodora.duca@email.umfiasi.ro (Ș.T.D.); dan-alexandru.costache@umfiasi.ro (A.D.C.); cepoi_maria-ruxandra@d.umfiasi.ro (M.-R.C.); sabinaandreea-leanca@email.umfiasi.ro (S.A.L.); irina.costache@umfiasi.ro (I.I.C.-E.); 2 Cardiology Clinic, “St. Spiridon” County Emergency Hospital, 700111 Iasi, Romania; crisanadrian93@yahoo.com; 3 III Internal Medicine Clinic, “St. Spiridon” County Emergency Clinical Hospital, 1 Independence Boulevard, 700111 Iasi, Romania; 4 Department of Microbiology, Faculty of Medicine, “Grigore T. Popa” University of Medicine and Pharmacy, 700115 Iasi, Romania; cristina.tuchilus@umfiasi.ro; 5 Microbiology Laboratory, “St. Spiridon” County Emergency Hospital, 700111 Iasi, Romania; 6 Department of Immunology, Faculty of Medicine, “Grigore T. Popa” University of Medicine and Pharmacy, 700115 Iasi, Romania; corina.cianga@umfiasi.ro (C.M.C.); daniela.constantinescu@umfiasi.ro (D.C.); 7 Immunology Laboratory, “St. Spiridon” County Emergency Hospital, 700111 Iasi, Romania; 8 Department of Orthopedics and Traumatology, Faculty of Medicine, “Grigore T. Popa” University of Medicine and Pharmacy, 700115 Iasi, Romania; awad.dmour@d.umfiasi.ro; 9 Cardiovascular Rehabilitation Clinic, Clinical Rehabilitation Hospital, 700661 Iasi, Romania; 10 Department of Medicine, Division of Cardiovascular Medicine, UTHealth, University of Texas Health Science Center at Houston, McGovern Medical School, Houston, TX 77225, USA; catalin.loghin@uth.tmc.edu; 11 Department of Morpho-Functional Sciences II, Faculty of Medicine, “Grigore T. Popa” University of Medicine and Pharmacy, 700115 Iasi, Romania; ionela.serban@umfiasi.ro

**Keywords:** endothelin-1, acute heart failure, biomarker, diagnosis, prognosis, multimorbidity

## Abstract

The management of acute heart failure (AHF) is becoming increasingly complex, especially in patients with multiple comorbidities. Endothelin-1 (ET-1), a vasoconstrictive peptide, is an important mediator of neurohormonal activation, endothelial dysfunction, and cardiac remodeling—key processes involved in the pathogenesis of AHF. The aim of our study was to evaluate the diagnostic and prognostic performance of ET-1 in multimorbid AHF patients, compared to established markers such as amino terminal pro B-type natriuretic peptide (NT-proBNP) and high-sensitivity cardiac troponin I (hs-cTnI). We conducted a single-center prospective study including 76 patients; 54 with AHF and 22 serving as controls. Upon admission, all patients underwent a comprehensive clinical, echocardiographic, and laboratory evaluation, including plasma ET-1 measurement using the enzyme-linked immunosorbent assay (ELISA) method. Receiver operating characteristic (ROC) curve and area under the curve (AUC) analysis were performed to assess the diagnostic and prognostic performance of ET-1 in comparison to NT-proBNP and hs-cTnI. ET-1 levels were considerably higher in AHF patients than in controls (*p* = 0.02), with an AUC of 0.954, showing comparable diagnostic accuracy with NT-proBNP (AUC = 0.997), alongside strong correlations with signs of systemic congestion, increased hospital stay, and ventricular dysfunction. ET-1 had the strongest predictive accuracy for in-hospital mortality (AUC = 0.781, *p* = 0.026), outperforming NT-proBNP and hs-cTnI. For 30-day mortality, ET-1 remained a reliable predictor (AUC = 0.784, *p* = 0.016). However, as the follow-up period extended to one year, its predictive power declined, confirming ET-1’s prognostic efficacy only for short-term outcomes. Moreover, ET-1 levels were not influenced by the presence of comorbidities, demonstrating its potential as an independent biomarker. Our findings support that ET-1 is a valuable biomarker for both diagnosis and short-term prognosis in the assessment of multimorbid AHF patients.

## 1. Introduction

Heart failure (HF) is a serious global health concern that has a significant impact on patient quality of life and healthcare systems due to its high morbidity and mortality rates. Its complex and heterogeneous nature, caused by various etiologies, pathophysiological mechanisms, and therapeutic responses, makes clinical management highly challenging. Traditional approaches that rely only on clinical assessments and standard biomarkers such as natriuretic peptides (NPs) frequently fail to address the complete spectrum of HF. Therefore, more comprehensive and individualized strategies are needed [1,2].

Acute heart failure (AHF) is a life-threatening syndrome that can occur suddenly in patients with no prior history of HF or manifest as an acute decompensation of previously stable chronic heart failure (CHF). AHF is one of the leading causes of emergency department visits. It is associated with poor clinical outcomes, such as high rates of hospital readmission and mortality, highlighting the importance of prompt diagnosis and efficient management [3,4].

The initial assessment of a patient with suspected AHF involves reviewing their cardiovascular history. It is also important to identify potential precipitating factors and rule out other pathologies that can have a similar presentation. Diagnostic confirmation is based on a systematic strategy that includes clinical examination, imaging, and biomarker evaluation [5,6,7].

NPs testing is essential for diagnosing and managing AHF. This is supported by major guidelines from the American College of Cardiology/American Heart Association (ACC/AHA) [8] and the European Society of Cardiology (ESC) [7]. Elevated concentrations of B-type natriuretic peptide (BNP ≥ 100 pg/mL), N-terminal pro-BNP (NT-proBNP ≥ 300 pg/mL), or mid-regional pro-atrial natriuretic peptide (MR-proANP ≥ 120 pg/mL) are strong indicators of AHF. In contrast, readings below these levels have a significant negative predictive value, meaning NPs are effective in ruling out AHF as a diagnosis. Besides diagnosing, these biomarkers are also important for predicting outcomes. They help guide treatment methods and contribute to the prevention of left ventricular (LV) dysfunction or new-onset HF [1]. Although NPs are highly sensitive for the identification of AHF, they lack specificity. Their levels can be influenced by numerous conditions beyond cardiac dysfunction, such as systemic inflammation, sepsis, renal impairment, obesity, pulmonary embolism, arrhythmias, and stroke [9]. Recently, an age-stratified approach using NT-proBNP has been proposed to improve diagnostic accuracy. This method considers how age influences biomarker levels, with specific cut-offs such as 450 pg/mL for those under 50, 900 pg/mL for those aged 50 to 75, and 1800 pg/mL for those over 75 [10].

However, given the complex nature of HF, relying on a single biomarker may not fully capture the disease’s pathophysiological heterogeneity [11]. Studies have demonstrated that high-sensitivity cardiac troponin (hs-cTn) holds both diagnostic and prognostic significance in AHF. It can predict all-cause mortality and outcomes during and after hospital stays [12,13,14,15]. On the other hand, hs-cTn is not disease specific, as elevated levels can also be seen in myocardial ischemia and chronic myocardial injury related to HF [11,16].

Furthermore, patients with AHF often present with multiple cardiovascular and non-cardiovascular comorbidities, which have a significant impact on prognosis and treatment strategies. The most common cardiovascular conditions are hypertension (~70%), coronary artery disease (~50–60%), and atrial fibrillation (~30–40%). Non-cardiovascular comorbidities such as diabetes (~40%), renal dysfunction (20–30%), and anemia (15–30%) further complicate the disease and worsen outcomes [17].

The burden of multimorbidity, defined as two or more chronic medical conditions, is especially high in HF patients. Prevalence rates range from 43% to 98%. Multimorbidity is strongly correlated with increased 1-year all-cause mortality, highlighting the importance of comprehensive care. With the growing number of elderly patients with comorbid conditions, frequent screening and early interventions are crucial. The ESC guidelines recommend a systematic evaluation of comorbidities in all suspected HF cases, acknowledging their importance in disease progression, quality of life, and long-term prognosis [18].

Diagnosing acute heart failure (AHF) is challenging due to complex pathophysiological mechanisms and the presence of multiple comorbidities [19]. The field of biomarkers in AHF is advancing rapidly, with several emerging markers showing potential to improve diagnosis and prognosis. Among these, galectin-3, mid-regional pro-adrenomedullin, growth differentiation factor-15 (GDF-15), soluble suppression of tumorigenicity 2, copeptin, and endothelin-1 (ET-1) are important. They are linked to key pathophysiological processes of AHF, such as cardiac remodeling, fibrosis, inflammation, neurohormonal activation, and endothelial dysfunction [20].

The discovery of ET-1 by Yanagisawa et al. in 1988 [21] was a milestone in cardiovascular science. ET-1 is the strongest endogenous vasoconstrictor discovered to date. This 21-amino acid peptide, primarily secreted by vascular endothelial cells and cardiomyocytes, is essential to vascular homeostasis, cardiac performance, and neurohormonal regulation. Its effect extends beyond vasoconstriction, influencing inflammation, oxidative stress, fibrosis, and ventricular remodeling, all of which are involved in the pathophysiology of acute and chronic HF [22,23,24].

ET-1 levels correlate with functional capacity and HF severity, with significantly higher concentrations observed in patients with moderate-to-severe HF compared to those with milder symptoms [25]. The ASCEND-HF (Acute Study of Clinical Effectiveness of Nesiritide in Decompensated Heart Failure) trial highlighted ET-1 as a prognostic marker in AHF. Higher baseline levels were associated with increased in-hospital adverse events and higher three-month mortality [26]. In addition, its course during hospitalization seems to be clinically significant, as greater reduction in ET-1 levels is associated with better outcomes. Likewise, the PROTECT trial, which evaluated 1653 patients with AHF, identified ET-1 as the best biomarker in predicting high-risk patients prone to post-discharge death or rehospitalization, further supporting its role in risk stratification [27]. Moreover, ET-1 also holds potential for guiding treatment strategies, offering a target for personalized care [28].

The accumulating evidence highlights ET-1 as a promising biomarker in AHF, with strong potential for clinical application. Our study aimed to evaluate the diagnostic and prognostic significance of ET-1 in comparison to conventional cardiac biomarkers, NT-proBNP and hs-cTnI, in patients with AHF and multimorbidities.

## 2. Materials and Methods

### 2.1. Study Design and Population Characteristics

This prospective study enrolled 76 patients admitted to the Cardiology Clinic at “St. Spiridon” Emergency County Hospital in Iași, Romania, between February and May 2023. Of these, 54 patients formed the AHF group, while the control group included 22 age- and sex-matched individuals who either had no history of HF or presented with stable, compensated CHF, without any recent decompensation or hospitalization. These individuals were admitted for non-specific cardiovascular complaints such as mild hypertension, palpitations, or chest discomfort. Patients presenting to the emergency department with sudden or rapidly worsening dyspnea were included in the AHF group if they were diagnosed with AHF according to the European Society of Cardiology criteria [7]. This diagnosis covered a range of HF presentations, including acute decompensated HF, cardiogenic shock, acute pulmonary edema, and isolated right ventricular failure.

For diagnosing AHF in patients presenting with acute dyspnea, the Framingham criteria were applied. Major criteria included signs of acute pulmonary edema on radiographic imaging, cardiomegaly with a cardiothoracic ratio greater than 0.5 on chest X-ray, paroxysmal nocturnal dyspnea, orthopnea, jugular venous distension, hepatojugular reflux, pulmonary rales, a third heart sound (gallop rhythm), and significant weight reduction (more than 4.5 kg) within five days of treatment. Minor criteria included the presence of ankle edema, exertional dyspnea, hepatomegaly, nocturnal cough, pleural effusion, and a heart rate above 120 beats per minute. To confirm the diagnosis, either two major criteria or a combination of one major and two minor criteria were required.

Individuals under 18 years old, pregnant women, and those with terminal malignancies or end-stage renal disease were excluded from both groups, along with patients with known neuropsychiatric disorders.

The study protocol received approval from the Ethics Committees of “Grigore T. Popa” University of Medicine and Pharmacy and “St. Spiridon” Emergency Clinical Hospital in Iași. This research was conducted following the principles outlined in the 2013 revision of the Declaration of Helsinki [29], with all participants, including those in the control group, providing informed written consent prior to inclusion.

### 2.2. Data Collection and Diagnostic Tests

To ensure a comprehensive evaluation, we conducted a detailed anamnesis and standard physical examination, followed by a meticulous review of the patients’ medical records. A thorough assessment of patients’ demographic characteristics, lifestyle habits, and comorbidities was conducted as part of the study. Detailed information was gathered through comprehensive anamneses, review of personal medical files, and hospital archives. Sociodemographic data included age and gender while lifestyle factors such as tobacco use, exposure to toxins, and alcohol consumption were carefully documented. Blood pressure and heart rate were recorded at the time of admission in the Cardiology Clinic.

Pre-existing conditions and those newly identified during hospitalization were systematically documented based on validated diagnostic criteria, focusing on conditions with significant cardiovascular implications.

All patients underwent a complete standard laboratory workup within the first hours of admission to the Cardiology Clinic. This included the measurement of established biomarkers, such as NT-proBNP and hs-cTnI, following current clinical guidelines. The pathological thresholds considered were NT-proBNP levels >125 pg/mL and hs-cTnI levels >29 ng/L. Comprehensive clinical data were supplemented with laboratory findings, including hemoglobin, hematocrit, sodium, potassium, renal function markers (urea, creatinine, bicarbonate, and estimated glomerular filtration rate [eGFR]), liver function markers (aspartate transaminase [AST], alanine transaminase [ALT], gamma-glutamyl transferase [GGT], alkaline phosphatase [ALP], total bilirubin, direct bilirubin), total serum proteins, and albumin. Additional parameters included glycemia, serum iron, ferritin, magnesium, inflammatory markers such as C-reactive protein (CRP), lactate dehydrogenase (LDH), creatine kinase (CK), creatine kinase myocardial band (CK-MB), uric acid, thyroid-stimulating hormone (TSH), cholesterol profile, and GDF-15.

The ET-1 and GDF-15 measurements were made on the same blood samples drawn during routine testing within the first 48 h of admission. Biological samples were collected through standard venipuncture into tubes containing potassium EDTA. To preserve the integrity of the samples, centrifugation was performed promptly (15 min at 1600× *g* at 0 °C) within 30 min of collection. Plasma was then aliquoted into sterile containers and stored at −80 °C until further analysis. ET-1 levels were quantified using a sandwich enzyme-linked immunosorbent assay (ELISA) (ET-1 ELISA Kit, Abcam 133030, Cambridge, UK). The assay’s detection range was 0.78–100 pg/mL, with a sensitivity of 0.41 pg/mL. The quantification of GDF-15 was performed using ELISA EIAab E2034h kits (EIAAB Science Inc., Wuhan, China), with a detection range of 15–1000 ng/L. All analyses were performed in a single batch to minimize variability, in the Immunology Laboratory of “St. Spiridon” Emergency Clinical Hospital.

Echocardiographic evaluation was performed on all patients included in the study upon admission.

### 2.3. Statstical Analysis

The Kolmogorov–Smirnov test was applied to assess the normal distribution of continuous variables within the study population. Parameters with a normal distribution are presented with minimum, maximum, and mean values ± standard deviation. For comparisons of mean values between groups for continuous variables, we employed the independent sample *t*-test. Categorical variables are expressed as frequencies and percentages. Differences between the AHF and control groups were analyzed using the independent sample *t*-test, with statistical significance set at a 5% threshold (*p* < 0.05). Additionally, paired sample statistics and paired *t*-tests were used to compare parameter values within patient groups.

Correlations between two variables were analyzed using Pearson and Spearman correlation coefficients (r), with the Pearson test for continuous variables and the Spearman test as a non-parametric alternative. Statistical significance was set at *p* < 0.05. Eta coefficients and ANOVA tests were used to quantify relationships between nominal and continuous variables by comparing mean values within each category. Graphical representations were used to illustrate the distributions of values across the study groups.

Linear regression was performed to evaluate and represent linear relationships between correlated variables, providing predictive value through the equation y = ax + b (where y is the dependent and x the independent variable). The regression coefficient was denoted by the slope of the regression line.

The diagnostic performance of biomarkers for AHF was evaluated using receiver operating characteristic (ROC) curve analysis, with area under the curve (AUC) comparisons. ST2 cut-off values were also derived from the ROC curve. We also performed a multivariate analysis to assess the independent predictive value of ET-1 for in-hospital and 30-day mortality, adjusting for age, NT-proBNP, and creatinine. Data analysis was conducted using IBM SPSS Statistics for Windows, version 26, and Microsoft Excel 2019 version 2502, for data organization prior to statistical processing. All statistical tests were two-tailed, and a *p*-value < 0.05 was considered statistically significant.

## 3. Results

### 3.1. Baseline Characteristics

This study included 76 participants, 54 with AHF and 22 in the control group. The mean age was similar between groups (67 ± 12 years vs. 66 ± 12 years, *p* = 0.58), with comparable gender distributions (72.2% males in AHF vs. 72.7% in controls, *p* = 0.98). Lifestyle factors such as smoking and alcohol consumption showed no significant differences. Smoking prevalence was 14.8% in AHF patients and 27.3% in controls, while alcohol abuse was reported equally in both groups (50%, *p* = 1.0). Clinical findings revealed significantly lower oxygen saturation (92 ± 4% vs. 96 ± 2%, *p* < 0.01) and higher respiratory rates in the AHF group (15 ± 2 vs. 14 ± 1 breaths per minute, *p* = 0.04) highlighting the resemblance between AHF and respiratory pathologies. As expected, signs of congestion, including pulmonary rales (68.5% vs. 4.5%, *p* < 0.001), pleural fluid (46.3% vs. 9.1%, *p* < 0.01), and bilateral limb edema (63% vs. 27.3%, *p* < 0.01), were more frequent in AHF patients. These findings underscore the clinical severity of the AHF group compared to controls (Table 1).

The laboratory findings revealed significant differences between the AHF group and the control group, highlighting key biomarkers associated with disease severity (Table 2). NT-proBNP levels were markedly elevated in the AHF group (7141 ± 6097 pg/mL) compared to controls (234 ± 210 pg/mL, *p* < 0.01), underscoring its role as a primary biomarker in AHF. Similarly, hs-cTnI levels were higher in AHF patients (3064 ± 1093 ng/L) than in controls (38.9 ± 0 ng/L, *p* = 0.19), suggesting a trend toward increased myocardial injury. Additionally, CRP was elevated in the AHF group compared to controls (*p* = 0.04), suggesting a heightened inflammatory state.

ET-1 concentrations were higher in the AHF group (5.28 ± 1.51 pg/mL) than in the control group (2.33 ± 0.38 pg/mL, *p* = 0.02), further supporting its potential as a diagnostic and prognostic biomarker. Renal function was notably impaired in AHF, with significantly higher creatinine levels, urea, and uric acid (*p* < 0.05).

Nutritional and metabolic markers also showed significant differences, with lower albumin and total protein levels in AHF patients (*p* < 0.01). Iron levels were reduced in AHF (53 ± 30 µg/dL vs. 87 ± 38 µg/dL, *p* < 0.01), indicating possible anemia and iron metabolism dysfunction. Lipid profile abnormalities were prominent, with elevated total cholesterol and LDL cholesterol (*p* < 0.01). These findings demonstrate the systemic impact of AHF, with significant changes in cardiovascular, inflammatory, renal, and metabolic biomarkers, reflecting the disease’s complexity and severity.

The analysis of comorbidities among study participants revealed that pulmonary hypertension was exclusively observed in the AHF group (*p* = 0.01), and atrial fibrillation was significantly more prevalent in AHF patients (50%) compared to controls (*p* = 0.01). Other frequent comorbidities included chronic kidney disease, anemia, dyslipidemia, diabetes mellitus type 2, hypertension, and chronic coronary syndrome, although without statistical significance (Table 3).

The echocardiographic evaluation highlighted significant cardiac remodeling and dysfunction in patients with AHF compared to the control group (Table 4). Both left and right atrium dilatation were significantly more prevalent in the AHF group, reflecting elevated atrial pressures and volume overload (0 < 0.01). AHF patients had a significantly larger right ventricle compared to controls (*p* = 0.01). Moreover, left ventricular ejection fraction (LVEF) was markedly reduced in AHF patients (*p* < 0.01) indicating severe systolic dysfunction as a hallmark of AHF.

**Figure 1:**
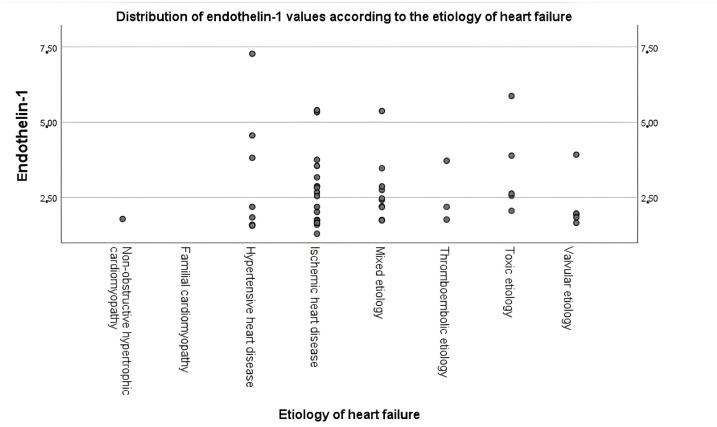
Distribution of ET-1 values according to the etiology of HF.

Given the wide range of etiologies in AHF, we analyzed ET-1 levels across different HF subtypes using etasquared coefficients and ANOVA tests. The results revealed significant differences in ET-1 levels across different etiologies of HF (*p* = 0.008), indicating that the mean values of ET-1 vary significantly between the groups. The F statistic (F = 3.147) highlights the variance observed between and within the groups, further supporting the statistical significance of the findings. The eta value of 0.569 suggests a moderate to strong association between ET-1 levels and the etiology of HF, as eta values between 0.41 and 0.60 are considered moderate, while those above 0.60 indicate a strong relationship. An eta squared of 0.324 demonstrates that approximately 32.4% of the variation in ET-1 levels can be attributed to differences in HF etiology, underscoring its potential role as a biomarker in distinguishing among HF subtypes (Figure 1, Table 5).

### 3.2. ET-1 Plasmatic Levels and Comorbidities

We performed a correlation analysis between ET-1 levels and the most common comorbidities in AHF patients as represented in Table 6. No significant associations were found, suggesting that ET-1 concentrations are not markedly influenced by the presence of common cardiovascular and non-cardiovascular conditions. The *p*-values across most common comorbidities in AHF indicate a lack of statistical significance, reinforcing ET-1’s potential as an independent biomarker.

**Figure 2:**
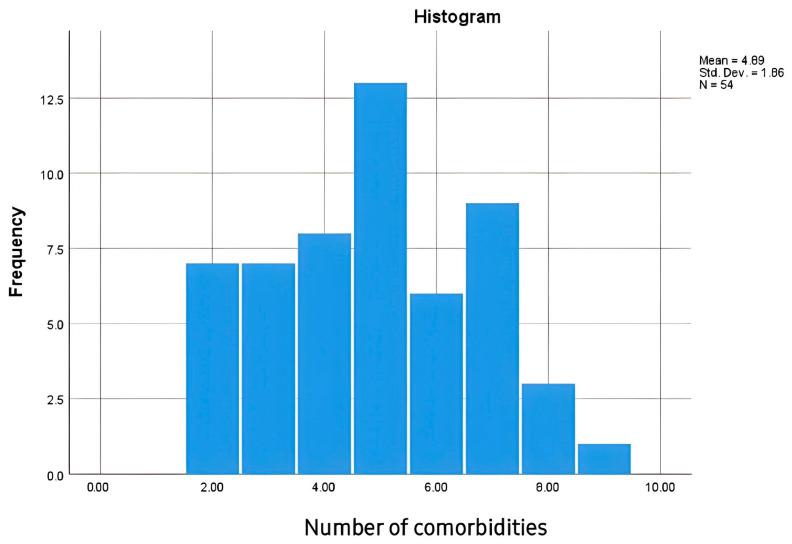
Distribution of comorbidities among study participants with AHF.

Multimorbidity is highly prevalent in HF, complicating management and contributing to worse clinical outcomes. In our study, patients with AHF had between two and nine comorbidities, highlighting the complex clinical profile of this population (Figure 2). Furthermore, our analysis did not reveal a statistically significant correlation between the number of comorbidities and ET-1 levels (R = 0.034, *p* = 0.819), suggesting that ET-1 concentrations are not substantially influenced by the presence of multiple comorbid conditions in studied population.

Furthermore, we analyzed the relationship between ET-1 levels and key clinical, biochemical, and echocardiographic parameters in AHF (Table 7). Our findings revealed significant associations between ET-1 and markers of congestion, ventricular dysfunction, and neurohormonal activation.

ET-1 levels were positively correlated with markers of congestion, including limb edema (R = 0.58, *p* < 0.01) and pulmonary rales (R = 0.35, *p* < 0.01), reinforcing its role in fluid retention and hemodynamic stress. Additionally, higher ET-1 levels were associated with prolonged hospitalization (R = 0.40, *p* < 0.01), suggesting a link between ET-1 and worse clinical outcomes. From a hemodynamic perspective, ET-1 exhibited an inverse correlation with systolic blood pressure (R = −0.31, *p* = 0.02) and a positive correlation with heart rate (R = 0.31, *p* = 0.02), indicating its involvement in vasoconstriction and neurohormonal activation. Additionally, a significant inverse correlation was observed with sodium levels (R = −0.38, *p* < 0.01). Moreover, ET-1 negatively correlated with LVEF (R = −0.28, *p* = 0.04) and TAPSE (R = −0.36, *p* < 0.01), highlighting its potential role in both left and right ventricular dysfunction. ET-1 was strongly correlated with NT-proBNP (R = 0.32, *p* = 0.01) and GDF-15 (R = 0.42, *p* < 0.01), further supporting its association with neurohormonal activation and systemic inflammation. Interestingly, no significant correlation was observed with hs-cTnI (R = −0.06, *p* = 0.62).

**Figure 3:**
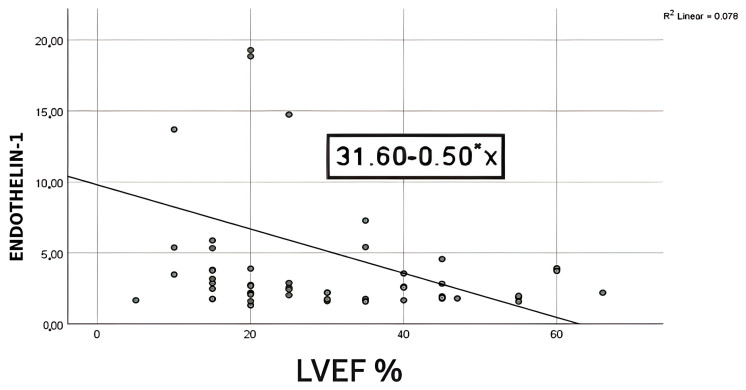
Simple scatter of ET-1 by LVEF.

Elevated ET-1 levels are associated with myocardial remodeling and fibrosis, processes that contribute to worsening cardiac function and reduced LVEF. We conducted a bivariate regression to examine how well the levels of ET-1 could predict the level of LVEF. A scatterplot showed that the relationship between ET-1 and LVEF was negative and linear and did not reveal any bivariate outliers (Figure 3). The correlation between ET-1 and LVEF was statistically significant (r = −0.28, *p* = 0.04). The regression equation for predicting LVEF from ET-1 was y = 31.60−0.50x. The r^2^ for this equation was 0.07; that is, 7% of the variance in LVEF was predicted by the level of ET-1. The bootstrapped 95% confidence interval for the slope to predict LVEF from ET-1 ranged from −0.98 to −0.02; thus, for each one unit of increase in ET-1, LVEF decreased by about 0.02–0.98 points (Table 8).

### 3.3. Diagnostic Accuracy of ET-1 in AHF

**Figure 4:**
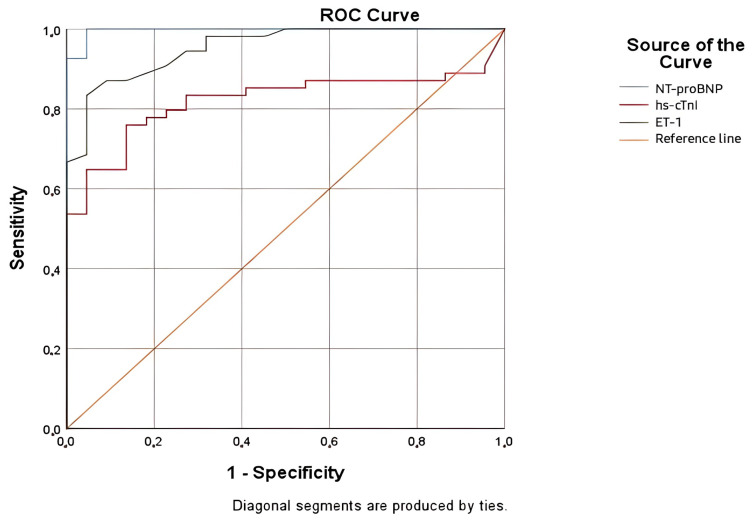
ROC curves for NT-proBNP, ET-1, and hs-cTnI for the diagnosis of AHF.

To evaluate the diagnostic accuracy of ET-1, a ROC curve analysis was conducted (Figure 4). ET-1 was an excellent diagnostic biomarker, with an AUC of 0.954, demonstrating high accuracy and reliability. Its strong performance was supported by a narrow confidence interval (95% CI: 0.911–0.997) and a statistically significant *p*-value (<0.001). This positions ET-1 as a valuable tool in clinical diagnostics, particularly when used alongside NT-proBNP or in situations where NT-proBNP results may be less clear due to external factors.

NT-proBNP remained the most accurate marker, with an almost perfect AUC of 0.997. However, ET-1 can offer a complementary role, broadening the diagnostic approach and adding robustness to clinical evaluations. In contrast, hs-cTnI, with an AUC of 0.820, showed moderate accuracy and was less reliable as a standalone marker (Table 9).

The ROC analysis for ET-1 demonstrated a threshold value of ≥1.68 pg/mL, with a sensitivity of 87.0% and a specificity of 86.4%. These findings highlight the potential diagnostic utility of ET-1, though further validation is required to determine its clinical applicability.

### 3.4. Prognostic Value of ET-1 in AHF

Beyond its diagnostic utility, previous studies have demonstrated the prognostic value of ET-1 in AHF. Therefore, we investigated its short- and long-term prognostic significance by performing ROC curve and AUC analysis to evaluate the relationship between ET-1, NT-proBNP, and hs-cTnI in predicting in-hospital mortality, as well as mortality at 30 days, 3 months, 6 months, and 1 year.

**Figure 5:**
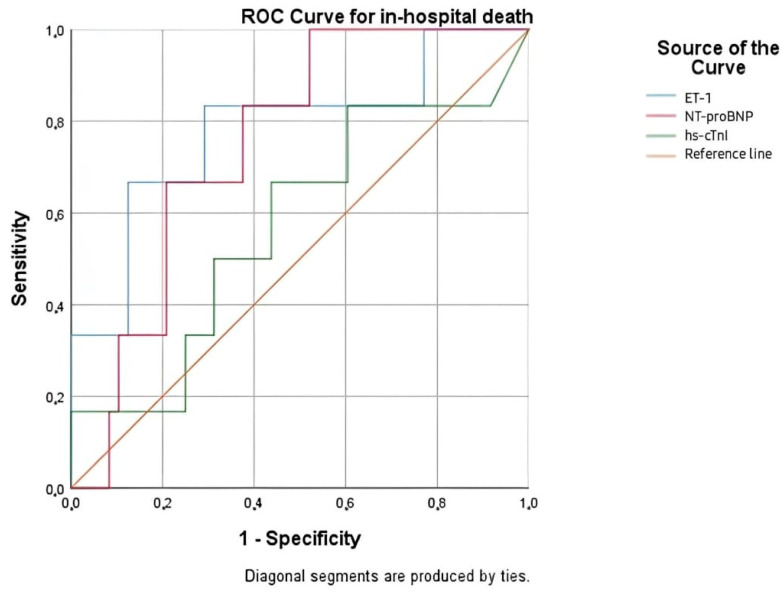
ROC curve for the relationship between cardiac biomarkers and in-hospital mortality rates.

The ROC curve analysis for in-hospital mortality demonstrated the prognostic value of ET-1, with an AUC of 0.781 (*p* = 0.026), followed by NT-proBNP with an AUC of 0.750 (*p* = 0.048). hs-cTnI, however, did not seem to provide significant predictive value in this context (AUC: 0.573, *p* = 0.563) (Figure 5, Table 10. ET-1 exhibited the highest predictive accuracy for in-hospital death. Thus, it is of particular interest to determine the optimal cut-off value of ET-1 for in-hospital mortality, as this could significantly contribute to risk assessment and early stratification of high-risk AHF patients. A cut-off value of 2.200 pg/mL for ET-1 was identified as the optimal threshold for predicting in-hospital mortality, demonstrating a sensitivity of 83.3% and a specificity of 43.7%.

**Figure 6:**
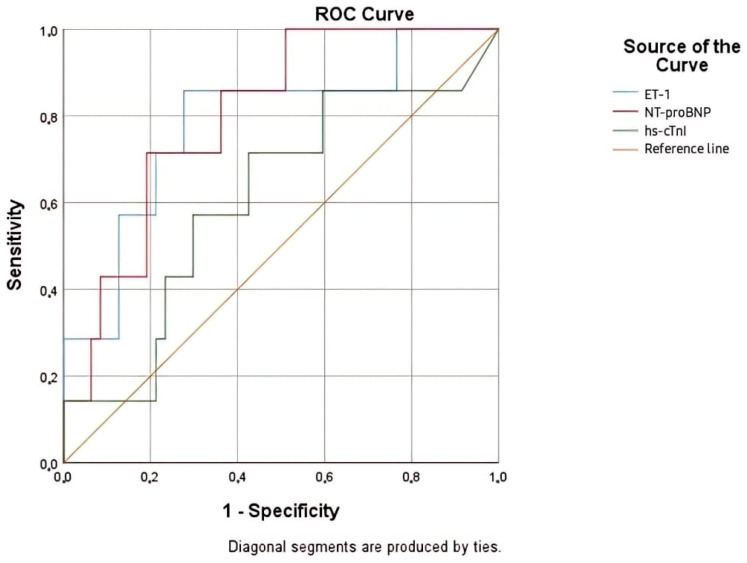
ROC curve for the relationship between cardiac biomarkers and 30-days mortality rate.

Compared to the results obtained for in-hospital mortality, where ET-1 showed the highest prognostic accuracy, NT-proBNP had the best performance in predicting 30-days mortality (AUC = 0.799), followed by ET-1 (AUC = 0.784). hs-cTnI could not be considered a reliable predictor (AUC = 0.611, *p*-value > 0.05) (Figure 6, Table 11).

**Figure 7:**
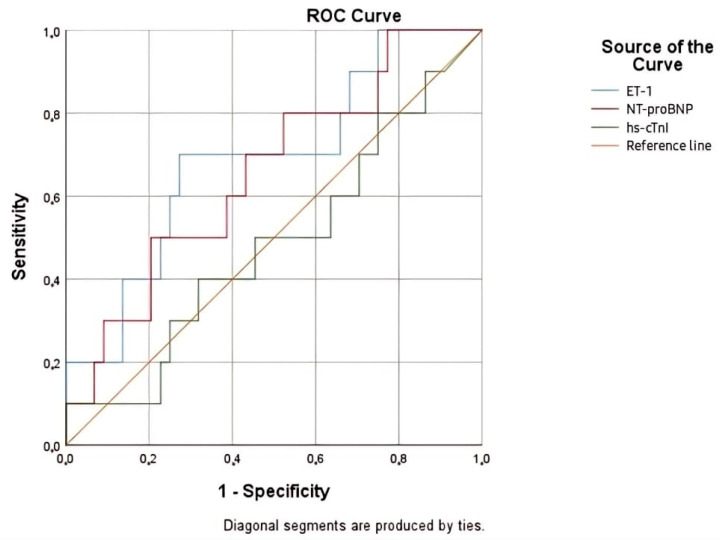
ROC curve for the relationship between cardiac biomarkers and 3 months mortality rate.

The evaluated biomarkers (ET-1, NT-proBNP, hs-cTnI) showed a decline in discriminatory capacity for mortality prediction as the follow-up period increased from 30 days to 3 months. Even if ET-1 had the highest AUC at 3 months (AUC 0.689, *p* 0.016), its lack of statistical significance limited its clinical utility for this time frame (Figure 7, Table 12). Additional variables may be needed to improve the prediction of mid-term mortality.

**Figure 8:**
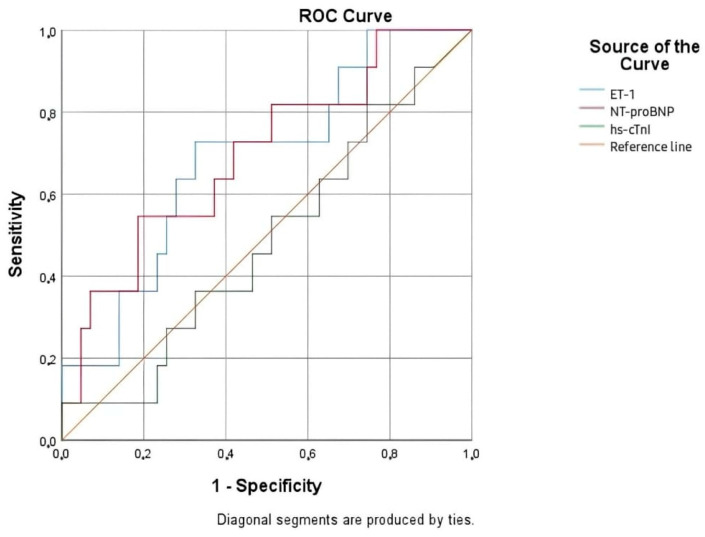
ROC curve for the relationship between cardiac biomarkers and 6 months mortality rate.

Compared to 3-months mortality, the biomarkers exhibited a similar predictive performance at 6 months. NT-proBNP remained the most reliable biomarker at this time point, although its discriminatory power was moderate, with an AUC below 0.7 (*p* > 0.05). ET-1 showed a comparable performance; however, its prognostic value was not statistically significant (*p* > 0.05). hs-cTnI remained a weak and clinically irrelevant predictor of mortality at both intervals (Figure 8, Table 13).

**Figure 9:**
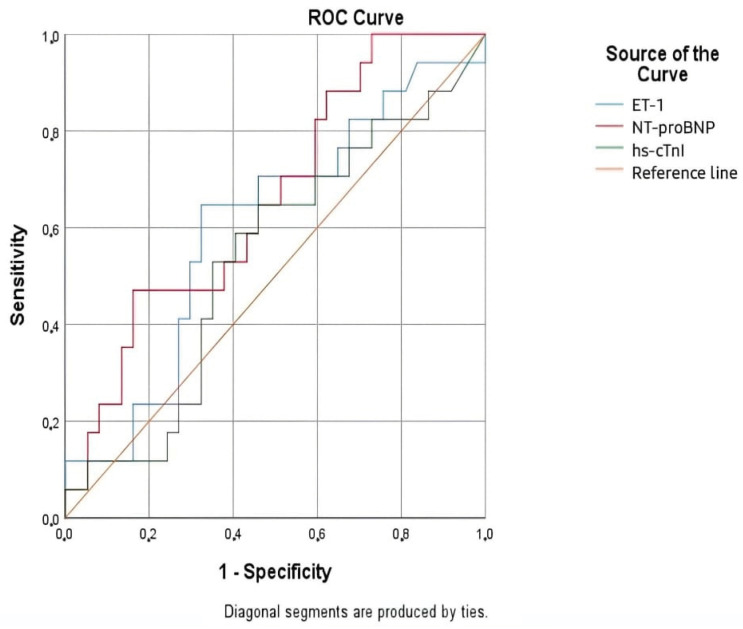
ROC curve for the relationship between cardiac biomarkers and 1-year mortality rate.

The analyzed biomarkers had limited capacity to predict 1-year mortality, as depicted in Figure 9. NT-proBNP remained the best-performing biomarker, although its statistical significance was marginal (AUC = 0.658; *p* = 0.064). ET -1 and hs-cTnI were not useful in this context for predicting long-term mortality (Table 14).

Given the established utility of ET-1 in short-term prognosis in AHF, we considered it relevant to construct a multimarker panel that includes age, NT-proBNP, ET-1, and creatinine, in order to determine their independent and combined predictive value for in-hospital and 30-days mortality. Multivariate analysis showed that the model was a significant predictor of in-hospital mortality (*p* < 0.01). Among the variables, ET-1 (*p* < 0.001) and creatinine (*p* = 0.032) were independent predictors, whereas age and NT-proBNP were not statistically significant (Table 15).

Similarly, the multivariate model analyzing 30-day mortality (Table 16) showed that ET-1 was still an independent, significant predictor (*p* < 0.001), while NT-proBNP had a borderline association (*p* = 0.052).

## 4. Discussion

AHF presents as a set of symptoms and clinical indications primarily caused by pulmonary and systemic congestion. Patients often experience dyspnea at rest or during activity. They may also report orthopnea, tiredness, and decreased exercise tolerance, which can significantly affect daily activities and overall quality of life. Physical examination typically reveals peripheral edema, jugular vein distension, pulmonary rales, and the presence of a third heart sound (S3 gallop). These findings indicate fluid overload and reduced cardiac function. Atypical or overlapping symptoms, particularly in multimorbid patients, often contribute to diagnostic errors. Studies have repeatedly demonstrated that delays in detection and management are associated with increased morbidity and death, emphasizing the necessity of rapid assessment and personalized treatment strategies [17,30]. Given these challenges, a comprehensive and multimodal diagnostic approach integrating clinical assessment, imaging, and biomarker measurement is required to improve diagnostic accuracy and patient care.

In recent years, biomarkers have gained importance for understanding the processes behind HF progression. Numerous studies have shown that neurohormonal activation is essential for cardiac function and HF pathophysiology. In response to the reduced cardiac output in HF, compensatory mechanisms such as neurohormonal activation are activated to maintain optimal blood pressure and perfusion to various important organs such as the brain, kidneys, and lungs. While these changes may help early on, they can negatively impact cardiac workload as HF worsens, eventually leading to acute decompensated HF (ADHF) [31,32]. Classical neurohormonal biomarkers include catecholamine levels, NPs, ET-1, and neprilysin [33]

The current guidelines strongly recommend the use of NPs for diagnosis, prognosis, and treatment guidance in HF. Furthermore, the AHA/ACC/HFSA guidelines uniquely support the use of additional biomarkers, such as galectin-3 and the soluble suppressor of tumorigenicity-2. NPs are important markers of hemodynamic stress in HF because they are generated in response to myocardial strain, elevated intracardiac pressures, chamber dilatation, and fluid overload. However, NPs levels are not exclusive to HF as they can be affected by a number of cardiovascular and non-cardiovascular diseases, leading to false elevations or suppressions [34].

ET-1, a vasoconstrictive peptide, implicated in a variety of aspects of HF pathogenesis including neurohormonal activation, endothelial dysfunction, cardiac remodeling, inflammation, and fibrosis, has emerged as a promising biomarker in AHF [35]. Multiple studies since its discovery have suggested that the degree of ET-1 is associated with the severity of symptoms and myocardial dysfunction in this pathology [3,35]. The purpose of our research was to assess whether ET-1 provides additional diagnostic and prognostic value beyond conventional biomarkers such as NT-proBNP and hs-cTnI in multimorbid AHF patients.

First of all, in our study, both the AHF cohort and the control group—which included people without HF or with stable, chronic HF—showed clinical characteristics that that closely align with the established pathophysiological concepts of HF. Compared to controls, the AHF group had reduced oxygen saturation and a greater respiratory rate. This likely reflected compromised gas exchange due to pulmonary congestion. AHF patients also had more signs of fluid overload, such as limb edema, pleural effusion, and pulmonary rales. Our findings revealed significant associations between ET-1 and the above-mentioned markers of congestion (*p* < 0.01), supporting its role in fluid retention and systemic congestion. Notably, the AHF group experienced longer hospital stays and higher costs, underlining the economic burden of this disease [36]. Additionally, in our study, higher ET-1 levels were related with age and prolonged hospitalization, indicating a possible interplay between ET-1 and adverse clinical outcomes.

The examined biomarkers (NT-proBNP, hs-cTnI and ET-1) showed higher levels in the AHF group. ET-1 levels were significantly and positively correlated with NT-proBNP. This supports the link to cardiac dysfunction, myocardial stretch, elevated filling pressure, and neurohormonal activation [33]. Measuring hs-cTn in AHF is indicated for diagnosing concurrent myocardial infarction type I as a triggering cause for HF decompensation and assessing cardiac myocyte damage. Even the absence of acute myocardial infarction, high levels of hs-cTn were associated with increased mortality and HF hospitalization [37,38]. Our study found that no significant connection was identified with hs-cTnI (r = −0.06, *p* = 0.62), suggesting that ET-1 may not directly indicate acute myocardial injury but rather reflects vascular and myocardial remodeling processes.

A novel biomarker that is receiving increasing attention in the medical literature is GDF-15. Also known as macrophage inhibitor cytokine-1, it is expressed by various cell types. These include cardiomyocytes, smooth muscle cells, and endothelial cells, especially in response to stress. GDF-15 is linked to reduced myocardial stress, ventricular remodeling, inflammatory, and apoptotic pathways. Although it is not a particular cardiac marker, elevated levels have been observed in HF [11,20]. GDF-15 is an excellent predictor of long-term mortality, surpassing NT-proBNP, the gold standard biomarker in HF [39,40]. For these reasons, we considered it appropriate to evaluate the relationship ET-1 and this biomarker. Our results demonstrated a strong correlation between ET-1 and GDF-15 (*p* < 0.01).

A key step in the development of HF is cardiac remodeling, which is characterized by changes to the structure and function of the heart. These alterations include decreased contractility, impaired systolic and diastolic function, and ultimately, heart enlargement and hypertrophy. The degree of remodeling significantly affects the clinical course of HF patients and is frequently associated with a worse prognosis [41]. Interestingly, neuroendocrine activation begins as soon as LV failure appears, even before HF manifests, and intensifies when clinical HF develops [41]. To evaluate the relationship between ET-1 levels and LVEF, we performed a bivariate regression. We discovered a strong negative association (r = −0.28, *p* = 0.04), indicating that LVEF falls as ET-1 levels rise, with ET-1 levels accounting for around 7% of the variation in LVEF. According to the bootstrapped 95% confidence interval for the slope (−0.98 to −0.02), there is a 0.02–0.88 point drop in LVEF for every unit rise in ET-1. The low r^2^ value (0.07) shows that ET-1 alone accounts for just a small part of the variation in LVEF. Therefore, it should be used in combination with other recognized markers for risk stratification. In line with this data, Perez et al. proved that ET-1 levels were significantly higher in those who presented with LVEF <50% [26]. Conversely, other studies have found no significant correlation between LVEF and ET-1 levels. Furthermore, they suggested that ET-1 is a significant predictor of unfavorable cardiovascular events and death, regardless of LVEF [26,42].

Numerous investigations have found higher ET-1 expression in the vascular endothelial cells of pulmonary arterial hypertension (PAH) patients. These patients also had raised ET-1 levels in the circulation. Additionally, PAH was one of the first disorders in which endothelin-targeting medicines were clinically tested [43]. TAPSE is recommended to be determined in all patients with PAH to assess right ventricle systolic function [44]. A total of 63% of the patients enrolled in our study had PAH. We observed a significant negative correlation between ET-1 and TAPSE, suggesting its role in mediating right ventricle dysfunction. Given the diverse causes of AHF, we examined ET-1 levels across various HF subtypes. Our analysis showed significant differences in mean ET-1 levels among the groups (*p* = 0.008). In fact, with an eta squared of 0.324, roughly 32.4% of the variability in ET-1 levels can be explained by the underlying etiology, suggesting that ET-1 could serve as a useful biomarker for distinguishing between HF subtypes. These findings highlight ET-1’s clinical importance in disease pathogenesis, as well as its potential use in risk stratification and treatment of patients with different HF etiologies.

Considering our current understanding of ET-1’s role in the pathophysiology of HF, we assumed that this neurohormone might be a valuable biomarker for the diagnosis and prognosis of AHF. This study provides new insights into the potential utility of ET-1 as a biomarker in HF, especially in comparison to traditional markers such as NT-proBNP and hs-cTnI. The evaluation of each biomarker and comparison were performed using an ROC curve and AUC, demonstrating excellent diagnostic accuracy for ET-1, with an AUC of 0.954. These results showed that the diagnostic performance of ET-1 is comparable with NT-proBNP, which remains the most accurate marker. Multiple studies had similar results, proving that ET-1 is elevated in AHF [26]. Based on ROC analysis for the diagnosis of AHF, we identified an ET-1 cut-off value of 1.68 pg/mL, with a sensitivity of 87.0% and a specificity of 86.4%.

In multimorbid patients, the diagnostic process is even more complex. The presence of various diseases such as atrial fibrillation, sepsis, chronic kidney disease, diabetes mellitus, pulmonary hypertension, and other acute or chronic pathologies can mask the classical clinical picture of AHF. This can lead to misinterpretation of the symptoms and diagnostic uncertainty. Furthermore, comorbidities influence both short- and long-term outcomes [45]. Our study’s prospective design, carried out in an emergency clinical hospital, made it possible to include patients with a variety of comorbidities alongside AHF.

The complexity of the clinical characteristics of AHF patients in our study was highlighted by the fact that they had between two and nine comorbidities. The number of comorbidities and ET-1 levels, however, did not significantly correlate. Moreover, our analysis revealed no significant relationship between ET-1 levels and the most common cardiac and non-cardiac comorbidities in AHF. This suggests that ET-1 concentrations are basically unaffected by these additional conditions, supporting its potential as a stand-alone biomarker. Previous studies indicated that ET-1levels are influenced by the presence of chronic kidney disease and systemic inflammation. However, we have found no statistically significant correlations between ET-1 and renal function or markers of inflammation such as CRP [46,47,48]. An explanation for this discrepancy is the specific patient population included in our study, since all patients were admitted to a specialized emergency cardiology ward and not to nephrology or infectious disease departments. Furthermore, our population was less likely to have severe renal impairment since patients with end-stage renal disease were specifically excluded. For this reason, most patients were not presenting with acute kidney injury or worsening of renal function at admission, which might have limited the disclosure of an association between ET-1 and renal dysfunction. In addition, in the setting of acute decompensated HF, hemodynamic changes, endothelial dysfunction, and neurohormonal activation could have had a more dominant influence on ET-1 levels compared with underlying renal impairment or inflammatory conditions.

Prior studies have demonstrated the prognostic value of ET-1 AHF. A study of 2359 patients in the Valsartan Heart Failure Trial provided additional evidence that a high ET-1 level is related with disease severity and can be regarded an independent prognostic factor [49]. According to another intriguing study that included 109 fully treated patients with HF From the evaluated parameters (ET-1 level, NYHA class, NT-proBNP, BNP level, LVEF, and age), only ET-1 had a significant and independent impact on prognosis. Moreover, this marker was able to identify a specific subgroup of patients who had an exceptionally high risk of mortality [25]. In the ASCEND-HF biomarker substudy, ET-1 gave more predictive information than NT-proBNP in hospitalized patients with AHF [26].

We analyzed the value of ET-1, NT-proBNP, and hs-cTnI for the prediction of in-hospital mortality, as well as mortality at 30 days, 3 months, 6 months, and 1 year. Besides its diagnostic value, ET-1 has provided significant prognostic value, particularly for the prediction of short-term mortality. The ROC analysis of in-hospital mortality in our study identified ET-1 as the most accurate predictor among the biomarkers investigated, with an AUC of 0.781 (*p* = 0.026), which was superior to NT-proBNP and significantly better than hs-cTnI. The optimal ET-1 cut-off level for in-hospital mortality was 2.200 pg/mL. ET-1 demonstrated good sensitivity (83.3%) but moderate specificity (43.7%), indicating its utility in the early risk stratification of severely ill AHF patients. In the prediction of 30-day mortality, NT-proBNP was the strongest biomarker, followed closely by ET-1. This supports that ET-1 remains a valid biomarker beyond the acute phase. However, as the follow-up period increased in length, the discriminatory power of all biomarkers, including ET-1, progressively declined. ET-1’s attenuation with prolonged follow-up may reflect its strong relationship with acute hemodynamic stress and endothelial activation—mechanisms especially significant during the early stages of decompensation.

In our multivariate analysis, the multimarker model that included age, creatinine, ET-1, and NT-proBNP showed promising predictive validity for 30-day and in-hospital mortality, highlighting the advantages of combining biomarkers to risk-stratify early in AHF. Even after controlling for biochemicals and clinical variables, ET-1 was the most reliable and powerful independent predictor, continuing to be statistically significant at both time points. Creatinine was also independently associated with in-hospital mortality but with diminishing prognostic value at 30 days. Age was not statistically significant, and NT-proBNP was marginally significant at 30 days and had no predictive value during hospital stay. Given its excellent performance in the short-term setting, ET-1 may serve as a useful early risk stratification marker, particularly when combined in a multimarker model with NT-proBNP, age, and creatinine for the identification of high-risk patients who require closer monitoring and more intensive medical approaches. Repeated or serial measurements of ET-1 over the course of hospitalization or follow-up may offer improved accuracy for long-term risk prediction and warrants further investigation in future research.

Recent studies, such as proteomic analysis by Gasparri et al. [50], have highlighted the importance of identifying new biomarkers for acute cardiovascular syndromes that can provide mechanistic data beyond myocyte injury. Similar to their findings in vitamin D-binding protein in STEMI, our study shows that ET-1 is not only a prognostic indicator but also a marker of vascular and neurohormonal stress in AHF. Interestingly, while hs-cTnI and NT-proBNP are markers of cardiac injury and wall stress, ET-1 appears to contribute to the overall pathophysiological background of AHF, particularly in multimorbid patients.

In the early stages of HF, the ET-1 elevation may be an adaptive mechanism used to preserve vascular tone and organ perfusion. However, as the disease progresses, this compensatory response becomes maladaptive. Persistent overexpression of ET-1 results in vasoconstriction, endothelial dysfunction, neurohormonal overdrive, myocardial fibrosis, and adverse cardiac remodeling, thus favoring further disease progression and poor outcomes. These findings are supported by the MESA Angiography Study [51], which investigated the prognostic value of ET-1 in a group without clinical cardiovascular disease. The investigators discovered that elevated ET-1 levels were associated with rising LVEF and falling LV end-diastolic volumes, implying a potential compensatory or protective role at the preclinical or early stages. However, in patients with established HF, increased ET-1 has repeatedly been associated with poor outcomes [52,53], supporting the idea that long-term ET-1 overexpression is detrimental. As a result, the ET-1 curve would most likely follow a biphasic trajectory; first compensatory, but eventually detrimental. Our results, with their high correlations between ET-1 and congestion indices, ventricular failure, and early mortality, validate its role as a maladaptive mediator in severe AHF, rather than an epiphenomenon.

Future research should explore whether serial ET-1 measurements could enhance risk stratification and guide treatment decisions, particularly in high-risk patients with persistent congestion despite optimal medical therapy. Given its strong association with neurohormonal activation and myocardial remodeling, ET-1 could be integrated into a multimarker approach, combining biomarkers that capture different aspects of AHF pathophysiology, such as myocardial stress (NT-proBNP), fibrosis and inflammation (GDF-15), and endothelial dysfunction (ET-1).

### Limitations of the Study

Despite the valuable information provided by this research, several limitations must be acknowledged. The primary limitation is the study’s single-center design and the small number of patients included. The small size of the control group reflects real-world patient recruitment limits in a tertiary cardiology emergency setting, where the vast majority of admissions are for acute cardiovascular conditions. This setting limited the number of clinically stable, non-decompensated individuals available for inclusion as controls. As a single-center prospective observational study, the findings reflect our center’s distinct patient population and care system, which may have an impact on wider applicability. Despite efforts to assure internal validity through strict matching and exclusion criteria, this imbalance may have an impact on the statistical power and generalizability of the results. Larger multicenter investigations are needed to validate the ET-1 diagnostic and prognostic significance in AHF. Another limitation is the absence of serial ET-1 measurements, which restricts our ability to evaluate its dynamic profile during hospitalization and follow-up. While ET-1 showed strong short-term prognostic value, its limited specificity and reduced performance over time indicate the need for additional variables and future studies incorporating repeated measurements to enhance long-term risk stratification. Finally, our study did not involve assessment of the response to therapy. Investigating how ET-1 levels correlate with therapeutic interventions (e.g., diuretics, inotropes, vasodilators) could offer valuable information on its role in guiding personalized treatment strategies. Future studies must discuss the inclusion of ET-1 in a multimarker approach and whether it can lead therapeutic intervention in high-risk AHF patients.

## 5. Conclusions

This study provides new insights into the diagnostic and prognostic value of ET-1 in multimorbid patients with AHF. Although NT-proBNP remains the gold standard biomarker, ET-1 showed comparable diagnostic accuracy, alongside strong correlations with signs of systemic congestion (bilateral limb edema, pulmonary rales, pleural effusion), as well as increased hospital stay and LV dysfunction. Beyond congestion, ET-1’s association with neurohormonal activation and cardiac remodeling was further supported by its strong correlation with NT-proBNP and GDF-15. Furthermore, we noted significant variations in ET-1 levels among HF etiologies, highlighting its emerging role in differentiating between HF subtypes.

In terms of prognostic performance, ET-1 outperformed NT-proBNP in terms of prediction accuracy for in-hospital mortality and continued to be a reliable indicator of 30-day post-discharge mortality. However, as the follow-up period extended to one year, its predictive power declined, confirming ET-1’s prognostic efficacy only for short-term outcomes.

Despite the high complexity of the study population, with patients having multiple comorbidities, our results showed that ET-1 levels were not influenced by the presence of these conditions, demonstrating its potential as an independent biomarker.

With its promise to reflect congestion severity, short-term mortality risk, and LV dysfunction, ET-1 exhibits strong potential as a diagnostic and prognostic biomarker in patients with multimorbid AHF. Additional research is needed to corroborate these findings and support the integration of ET-1 into clinical practice, where it could improve risk classification, guide therapeutic strategies, and optimize care in multimorbid AHF patients.

## Figures and Tables

**Table 1:** Baseline characteristics.

	Total (*n* = 76)			AHF (*n* = 54)			Control Group (*n* = 22)			
	Min	Mean ± STD	Max	Min	Mean ± STD	Max	Min	Mean ± STD	Max	*p* Value
**Age**	35	67 ± 12	91	35	67 ± 12	91	42	66 ± 12	83	0.58
**Male (N, %)**		55 (72.4%)			39 (72.2%)			16 (72.7%)		0.98
**Female (N, %)**		21 (27.6%)			15 (27.8%)			6 (27.3%)		
**Urban area of residence (N, %)**		46 (60.5%)			34 (63%)			12 (54.5%)		0.5
**Hospitalization days**	2	7.89 ± 6.54	56	3	8.56 ± 7.52	56	2	6.27 ± 2.47	10	0.16
**Smoker (N, %)**		14 (18.40%)			8 (14.8%)			6 (27.3%)		0.42
**Alcohol abuse (N, %)**		50 (50%)			27 (50%)			11 (50%)		1.0
**Toxic environment (N, %)**		13 (17.1%)			12 (22.2%)			1 (4.5%)		0.06
**Body mass index (kg, m^2^)**	17.30	28.01 ± 5.61	48.10	17.30	28.36 ± 6.19	48.10	21.80	27.14 ± 3.84	34.29	0.39
**Systolic blood pressure (mmHg)**	80	129 ± 20	220	80	128 ± 21	220	90	131 ± 19	175	0.68
**Diastolic blood pressure (mmHg)**	60	81 ± 12	110	60	80 ± 13	110	60	84 ± 12	105	0.26
**Heart rate (bpm)**	30	87 ± 27	160	33	90 ± 28	160	30	80 ± 24	150	0.15
**SpO2 (%)**	84	93 ± 4	99	84	92 ± 4	99	92	96 ± 2	99	<0.01
**Respiratory rate**	14	15 ± 2	20	14	16 ± 1	20	14	15 ± 1	18	0.04
**Bilateral limb edema (N, %)**		45 (83%)			39 (72.2%)			6 (27.3%)		<0.01
**Pleural fluid (N, %)**		27 (35.5%)			25 (46.3%)			2 (9.1%)		<0.01
**Pulmonary rales (N, %)**		45 (83%)			44(81.4%)			1 (4.5%)		<0.001

Legend: N—number; STD—standard deviation; SpO2—oxygen saturation; AHF—acute heart failure.

**Table 2:** Laboratory parameters.

	Total (n = 76)			AHF (n = 54)			Control Group (n = 22)			
	Min	Mean ± Std. Dev	Max	Min	Mean ± Std. Dev	Max	Min	Mean ± Std. Dev	Max	*p* Value
**NT-proBNP (pg/mL)**	25	5141 ± 6018	31,210	518	7141 ± 6097	31,210	25	234 ± 210	945	<0.01
**hs-cTnI (ng/L)**	0.05	2179 ± 9295	50,000	0.05	3064 ± 10932	50,000	0.05	7.07 ± 8.50	38.90	0.19
**GDF-15 (ng/L)**	83.51	665.57 ± 61.11	3257	191	631.34 ± 567.635	3257	83.51	321.35 ± 34.32	770	<0.01
**CK (mg/dL)**	22	305 ± 940	6467	22	381 ± 1107	6467	29	116 ± 107	484	0.26
**CK-MB (mg/dL)**	7	48.94 ± 147.50	1200	7	61.94 ± 173.73	1200	9	17.33 ± 5.28	28	0.23
**CRP (mg/dL)**	0.04	2.42 ± 3.38	12.20	0.07	2.46 ± 3.41	12.06	0.04	2.33 ± 3.40	12.20	0.88
**Endothelin-1 (pg/mL)**	0.27	4.10 ± 7.39	51.34	1.30	5.28 ± 8.51	51.34	0.27	1.21 ± 0.44	2.02	0.02
**Leukocyte (cmm)**	4770	8500 ± 2473	16,320	4770	8803 ± 2671	16,320	4900	7757 ± 1742	10.540	0.09
**Red cells (milion/yL)**	1.8	4.4 ± 1.1	9.9	1.8	4.4 ± 1.1	9.9	3.01	4.55 ± 0.57	5.61	0.76
**Hemoglobin (g/dL)**	8.0	13.26 ± 2.21	18.80	8.0	13.0 ± 2.34	18.80	9.8	13.88 ± 1.75	17.20	0.12
**Hematocrit (%)**	23.20	39.73 ± 6.19	59.10	23.20	39.17 ± 6.71	59.10	30.9	41.12 ± 4.53	49.6	0.21
**Glucose (mg/dL)**	60	121 ± 48	305	60	128 ± 52	305	81	106 ± 32	225	0.07
**Sodium (mmol/L)**	123	139 ± 4	147	123	138 ± 4	147	135	139 ± 2	144	0.45
**Potassium (mmol/L)**	2.7	4.5 ± 0.6	7.0	2.7	4.5 ± 0.6	7.0	3.4	4.4 ± 0.5	5.7	0.45
**Magnesium**	1.1	1.9 ± 0.3	4.0	1.1	2.0 ± 0.3	4.0	1.19	1.9 ± 0.2	2.7	0.25
**Alkaline reserve (mEq/L)**	14.70	23.57 ± 3.89	38.10	14.70	23.09 ± 4.14	38.10	19.70	24.76 ± 2.94	31.10	0.09
**Urea (mg/dL)**	20	55.04 ± 27.59	152	20	60.19 ± 30.0	152	20	42.41 ± 14.58	80	0.01
**Creatinine (mg/dL)**	0.58	1.1 ± 0.47	2.59	0.58	1.22 ± 0.50	2.59	0.62	0.98 ± 0.36	2.25	0.04
**GFR (Cockcroft Gault) mL/min/1.73 m^2^**	19.92	75.97 ± 35.60	174.25	19.92	72.75 ± 36.64	174.25	39.21	83.88 ± 31.33	169.30	0.21
**Uric acid (mg/dL)**	2.3	6.8 ± 2.5	17.8	2.3	7.3 ± 2.9	17.8	3.6	5.7 ± 1.3	8.0	0.01
**ALT (IU/L)**	10	56 ± 96	606	10	68 ± 112	606	12	26 ± 16	81	0.09
**AST (IU/L)**	7	51 ± 87	538	7	57 ± 96	538	8	36 ± 55	275	0.34
**GGT (IU/L)**	8	55 ± 54	286	12	62 ± 59	286	8	39 ± 35	130	0.09
**Direct bilirubin (mg/dL)**	0.10	0.41 ± 0.20	1.35	0.13	0.49 ± 0.27	1.31	0.10	0.33 ± 0.15	0.70	0.02
**Total bilirubin (mg/dL)**	0.19	0.96 ± 0.58	3.20	0.29	1.06 ± 0.63	3.20	0.19	0.73 ± 0.33	1.35	0.03
**Total protein (mg/dL)**	4.7	6.7 ± 0.7	8.8	4.7	6.5 ± 0.6	8.2	6.3	7.2 ± 0.5	8.8	<0.01
**Albumin (mg/dL)**	2.3	3.9 ± 0.5	5.0	2.34	3.8 ± 0.5	4.80	3.4	4.3 ± 0.3	5.0	<0.01
**LDH (U/L)**	93	300 ± 316	2540	93	340 ± 364	2540	123	195 ± 47	283	0.08
**TSH (mIU/L)**	0.01	2.12 ± 1.62	7.56	0.04	2.35 ± 1.75	7.56	0.01	1.49 ± 1.01	3.87	0.09
**Total Cholesterol (mg/dL)**	63	155 ± 46	266	63	144 ± 43	255	107	181 ± 43	266	<0.01
**LDL-cholesterol** **(mg/dL)**	42	104 ± 40	219	42	97 ± 38	196	74	124 ± 41	219	<0.01
**HDL-cholesterol (mg/dL)**	6	39 ± 14	109	6	36 ± 15	109	22	45 ± 12	71	0.01
**Triglycerides** **(mg/dL)**	42	109 ± 43	221	51	109 ± 45	221	42	109 ± 41	208	0.99
**Iron (µg/dL)**	10	63 ± 35	155	10	53 ± 30	155	22	87 ± 38	140	<0.01
**Ferritin (ng/mL)**	17	222 ± 287	2297	17	216 ± 325	2297	18	235 ± 166	612	0.79

Legend: AHF—acute heart failure; GDF-15—growth differentiating factor-15; NT-proBNP—amino-terminal pro-B-type natriuretic peptide. hs-cTnI—high-sensitive cardiac troponin I. CRP—reactive protein; LDH—lactate dehydrogenase; CK—creatine kinase; CK-MB—creatine kinase myocardial band; ALT—alanine transaminase; AST—aspartate transaminase; LDL—low density lipoprotein; HDL—high density lipoprotein cholesterol; TSH—thyroid stimulating hormone; GFR—Glomerular Filtration Rate.

**Table 3:** Comorbidities.

	Total (*n* = 76)	AHF (*n* = 54)	Control Group (*n* = 22)	*p*
**Chronic coronary syndrome (N, %)**	32 (42.1%)	24 (44.4%)	8 (36.4%)	0.35
**Hypertension (N, %)**	47 (61.8%)	33 (61.1%)	14 (63.6%)	0.79
**Chronic venous insufficiency (N, %)**	36 (47.4%)	26 (48.1%)	10 (45.5%)	0.8
**Infectious disease (N, %)**	17 (22.4%)	15 (27.8%)	2 (9.1%)	0.07
**Gastroenterological disease (N, %)**	43 (56.6%)	31 (57.4%)	12 (54.5%)	0.82
**Chronic kidney disease (n, %)**	40 (53.2%)	25 (46.3%)	15 (68.2%)	0.08
**Diabetes mellitus type 2 (N, %)**	24 (31.6%)	20 (37%)	4 (18.2%)	0.11
**Dyslipidemia (N, %)**	55 (72.4%)	38 (70.4%)	17 (77.3%)	0.54
**Anemia (N, %)**	27 (35.5%)	21 (38.9%)	6 (27.3%)	0.34
**Atrial fibrillation (N, %)**	31 (40.78%)	27 (50%)	4 (18.2%)	0.01

Legend: N—number, AHF—acute heart failure.

**Table 4:** Echocardiographic parameters.

	Total (*n* = 76)	AHF (*n* = 54)	Control Group (*n* = 22)	
	Mean ± STD	Mean ± STD	Mean ± STD	*p* Value
**Dilated left atrium (N, %)**	54 (73.3%)	43 (79.6%)	11 (50%)	0.008
**Dilated right atrium (N, %)**	56 (75%)	45 (83.3%)	11 (50%)	0.001
**Right ventricle (mm)**	35 ± 6	36 ± 6	32 ± 3	0.01
**LV end-diastolic diameter (mm)**	53 ± 10	54 ± 10	48 ± 8	0.01
**Interventricular septum (mm)**	11 ± 2	11 ± 2	11 ± 1	0.24
**Posterior wall of LV (mm)**	10 ± 1	10 ± 1	10 ± 1	0.68
**Maximum velocity in the aorta (m/s)**	1.53 ± 0.76	1.5 ± 0.8	1.46 ± 0.28	0.67
**LVEF (%)**	34.97 ± 17.42	28.94 ± 15.31	49.77 ± 13.04	<0.01

Legend: AHF—acute heart failure; LV—left ventricle; STD—standard deviation; LVEF—left ventricular ejection fraction.

**Table 5:** ET-1 values in different subtypes of HF.

			Sum of Squares	df	Mean Square	F	Sig.
ENDOTHELIN-1 × Etiology of HF	Between Groups	(Combined)	1243.008	7	177.573	3.147	0.008
	Within Groups		2595.995	46	56.435		
	Total		3839.003	53			
Measures of Association							
		Eta		Eta Squared			
ENDOTHELIN-1 × Etiology of HF		0.569		0.324			

Legend: df—degrees of freedom; F—F-statistic; Sig.—significance value.

**Table 6:** Correlations between ET-1 levels and comorbidities.

	*p*-Value	R
**Type 2 diabetes mellitus**	0.99	0.01
**Chronic kidney disease**	0.72	0.05
**Arterial hypertension**	0.08	0.23
**Chronic coronary syndrome**	0.34	0.12
**Anemia**	0.19	0.17
**Chronic venous insufficiency**	0.41	−0.11
**Atrial fibrillation**	0.09	−0.23
**Dyslipidemia**	0.48	−0.09
**Gastroenterological disease**	0.619	0.08
**Infectious disease**	0.18	0.18

**Table 7:** Correlations between ET-1 plasma levels and relevant parameters.

ET-1		
	*p*-Value	R
**Age**	0.01	−0.32
**Hospitalization days**	<0.01	0.40
**Systolic blood pressure**	0.02	−0.31
**Diastolic blood pressure**	0.66	0.06
**Heart rate**	0.02	0.31
**LV end-diastolic diameter**	0.84	0.02
**Posterior wall of LV**	0.13	−0.20
**Right Ventricle diameter**	<0.01	0.35
**Interventricular septum**	0.04	−0.27
**LVEF**	0.04	−0.28
**TAPSE**	<0.01	−0.36
**NT-proBNP**	0.01	0.32
**CK**	<0.01	0.54
**hs-cTnI**	0.62	−0.06
**CK-MB**	0.95	0.01
**CRP**	0.11	0.21
**GDF-15**	<0.01	0.42
**Leukocyte**	0.32	0.13
**Hemoglobin**	0.98	0.01
**Sodium**	<0.01	−0.38
**Potassium**	0.20	0.17
**Magnesium**	0.82	−0.03
**Alkaline reserve**	0.11	−0.21
**Uree**	0.75	0.04
**Creatinine**	0.79	−0.03
**Uric Acid**	0.43	0.10
**ALT**	0.06	0.25
**AST**	<0.01	0.25
**Total protein**	0.08	−0.23
**Albumin**	0.08	−0.24
**LDH**	0.74	0.04
**TSH**	0.07	0.28
**Total cholesterol**	0.09	−0.23
**LDL-cholesterol**	0.24	−0.16
**HDL-cholesterol**	0.08	−0.23
**Triglycerides**	0.83	−0.02
**Iron**	0.14	−0.20
**Ferritin**	0.28	−0.14
**Bilateral limb edema**	<0.01	0.58
**Pulmonary rales**	<0.01	0.35
**Pleural fluid**	0.03	−0.28

Legend: LV—left ventricle GDF-15-growth differentiating factor-15; NT-proBNP—amino-terminal pro-B-type natriuretic peptide; hs-cTnI—high-sensitive cardiac troponin I; CRP—reactive protein; LDH—lactate dehydrogenase; CK—creatine kinase; CK-MB—creatine kinase myocardial band; ALT—alanine transaminase; AST—aspartate transaminase; LDL—low density lipoprotein; HDL—high density lipoprotein cholesterol; TSH—thyroid stimulating hormone; LVEF—left ventricle ejection fraction; TAPSE—tricuspid annular plane systolic excursion.

**Table 8:** Correlation between ET-1 levels and LVEF in AHF patients.

Model		Unstandardized Coefficients		Standardized Coefficients	t	Sig.	95.0% Confidence Interval for B	
		B	Std. Error	Beta			Lower Bound	Upper Bound
**1**	(Constant)	31.604	2.383		13.262	0.000	26.822	36.386
	ENDOTHELIN-1	−0.503	0.240	−0.280	−2.102	0.040	−0.984	−0.023
**ANOVA**								
**1**	Model	Sum of Squares	df	Mean Square	F	Sig.		
	Regression	973.006	1	973.006	4.418	0.040		
	Residual	11,451.827	52	220.227				
	Total	12,424.833	53					

**Table 9:** Detailed analysis of AUC: diagnostic performance of ET-1 compared to NT-proBNP and hs-cTnI.

Test Result Variable(s)	Area Under the Curve	Std. Error	95% Confidence Interval		
			Lower Bound	Upper Bound	*p* Value
**ET-1**	0.954	0.022	0.911	0.997	<0.001
**NT-proBNP**	0.997	0.004	0.989	1.000	<0.001
**hs-cTnI**	0.820	0.048	0.726	0.915	<0.001

**Table 10:** Detailed analysis of the AUC: ET-1, NT-proBNP, and hs-cTnI correlation to in-hospital mortality rates.

Test Result Variable(s)	Area Under the Curve	Std. Error	*p* Value	95% Confidence Interval	
				Lower Bound	Upper Bound
**ET-1**	0.781	0.113	0.026	0.560	1.000
**NT-proBNP**	0.750	0.079	0.048	0.594	0.906
**Hs-cTnI**	0.573	0.130	0.563	0.318	0.828

**Table 11:** Detailed analysis of the AUC: cardiac biomarkers and 30-days mortality rate.

Test Result Variable(s)	Area Under the Curve	Std. Error	*p* Value	95% Confidence Interval	
				Lower Bound	Upper Bound
**ET-1**	0.784	0.099	0.016	0.591	0.977
**NT-proBNP**	0.799	0.075	0.011	0.652	0.947
**Hs-cTnI**	0.611	0.118	0.347	0.380	0.841

**Table 12:** Detailed analysis of the AUC: cardiac biomarkers and 3 months mortality rate.

Test Result Variable(s)	Area Under the Curve	Std. Error	*p* Value	95% Confidence Interval	
				Lower Bound	Upper Bound
**ET-1**	0.689	0.094	0.065	0.504	0.874
**NT-proBNP**	0.657	0.094	0.124	0.473	0.841
**hs-cTnI**	0.484	0.104	0.876	0.280	0.688

**Table 13:** Detailed analysis of the AUC: cardiac biomarkers and 6 months mortality rate.

Test Result Variable(s)	Area Under the Curve	Std. Error	*p* Value	95% Confidence Interval	
				Lower Bound	Upper Bound
**ET-1**	0.687	0.088	0.057	0.515	0.859
**NT-proBNP**	0.696	0.090	0.047	0.519	0.872
**hs-cTnI**	0.484	0.097	0.872	0.294	0.674

**Table 14:** Detailed analysis of the AUC: cardiac biomarkers and 1-year mortality rate.

Test Result Variable(s)	Area Under the Curve	Std. Error	*p* Value	95% Confidence Interval	
				Lower Bound	Upper Bound
**ET-1**	0.603	0.084	0.226	0.439	0.768
**NT-proBNP**	0.658	0.078	0.064	0.506	0.810
**hs-cTnI**	0.536	0.085	0.675	0.369	0.703

**Table 15:** Multivariate analysis for predictors of in-hospital mortality.

Model	Unstandardized Coefficients		Standardized Coefficients	t	Sig.	95.0% Confidence Interval for B	
	B	Std. Error	Beta			Lower Bound	Upper Bound
Multimarker panel	2.41	0.223		10.85	0.000	1.97	2.86
Age	−0.003	0.003	−0.129	−1.00	0.319	−0.010	0.003
NT-proBNP	−6.196 × 10^−7^	0.000	−0.012	−0.098	0.922	0.000	0.000
ET-1	−0.020	0.005	−0.534	−4.27	0.000	−0.029	−0.011
Creatinine	−0.168	0.076	−0.267	−2.20	0.032	−0.320	−0.015

**Table 16:** Multivariate analysis for predictors of 30-days mortality.

Model	Unstandardized Coefficients		Standardized Coefficients	t	Sig.	95.0% Confidence Interval for B	
	B	Std. Error	Beta			Lower Bound	Upper Bound
Multimarker panel	2.48	0.234		10.630	0.000	2.01	2.95
Age	−0.004	0.003	−0.136	−1.080	0.286	−0.010	0.003
NT-proBNP	−1.321 × 10^−5^	0.000	−0.237	−1.993	0.052	0.000	0.000
ET-1	−0.019	0.005	−0.471	−3.843	0.000	−0.029	−0.009
Creatinine	−0.145	0.080	−0.216	−10.825	0.074	−0.305	0.015

## Data Availability

The original contributions presented in this study are included in the article. Further inquiries can be directed to the corresponding authors.
